# Near-infrared excited luminescence and *in vitro* imaging of HeLa cells by using Mn^2+^ enhanced Tb^3+^ and Yb^3+^ cooperative upconversion in NaYF_4_ nanocrystals

**DOI:** 10.1039/c9na00336c

**Published:** 2019-07-12

**Authors:** Katarzyna Prorok, Michał Olk, Michał Skowicki, Agnieszka Kowalczyk, Agata Kotulska, Tomasz Lipiński, Artur Bednarkiewicz

**Affiliations:** Institute of Low Temperature and Structure Research, Polish Academy of Sciences Okolna 2 50-422 Wroclaw Poland k.prorok@intibs.pl; Institute of Immunology and Experimental Therapy, Polish Academy of Sciences R. Weigla 12 53-114 Wroclaw Poland; Lukasiewicz Research Network – PORT Polish Center for Technology Development Stablowicka 147 54-066 Wroclaw Poland

## Abstract

Advanced biodetection and bioimaging require fluorescent labels which exhibit many, easily distinguishable colors to identify or study numerous biotargets in a single sample. Although numerous different colors have been demonstrated with lanthanide doped nanoparticles, these colors usually originate from various ratios of overlapping multiple emission bands from activators, which severely limits the number of available labels. As a consequence, different lanthanide doped labels cannot be easily distinguished from each other (*e.g.* Er^3+^ from Ho^3+^) in a quantitative way, when such labels are co-localized during microscopy wide-field imaging. It is therefore reasonable to expand the available choice of spectral signatures and not rely on just different colors. Other ions, such as Tb^3+^ or Eu^3+^, can offer new possibilities and unique spectral features in upconversion mode in this respect. For example, despite partial overlap with Er^3+^ or Ho^3+^ emission spectra, Tb^3+^ ions display also unique and easily distinguishable spectral features at 580 nm. Unfortunately, in terms of brightness, Tb^3+^ emission in upconversion mode is typically too weak to be useful. To improve the Tb^3+^ upconversion emission intensity, a new approach, *i.e.* Mn^2+^ co-doping, has been proposed and verified in this work. A versatile optimization of Tb^3+^, Yb^3+^ and Mn^2+^ ion concentrations has been performed based on luminescence spectra and lifetime studies. The most intense emission was achieved for nanoparticles doped with 10% Mn^2+^ ions, with over 30 times brighter intensity of Tb^3+^ ions compared to the emission of nanocrystals without the addition of Mn^2+^ ions. Additionally, as a proof of the concept, the surface of nanoparticles was coated with proteins and conjugated with folic acid, and such biofunctionalized nanoparticles were subsequently used for bioimaging of HeLa cells.

## Abstract 2

To improve the Tb^3+^ upconversion emission intensity, a new approach, *i.e.* Mn^2+^ co-doping, has been proposed and verified in this work. The significant enhancement of the emission intensity as a result of the introduction of Mn^2+^ ions was observed.

## Graphical abstract

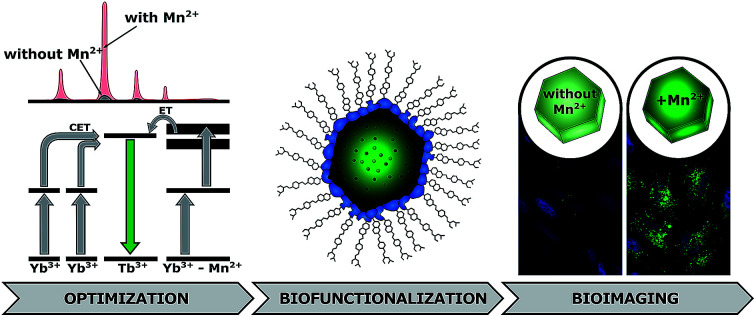

## 1 Introduction

Upconverting nanoparticles (UCNPs) have gained considerable interest as nano-colloidal biolabels in biodetection and bioimaging.^1–5^ However, some high-throughput detection/imaging schemes require the use of labels exhibiting many, distinguishable spectral fingerprints to identify or study many biotargets co-localized in a single sample. It is therefore reasonable to expand the available choice of color signatures. Many colors have been obtained with UCNPs based on ratiometric designs, where the content of one emitter changes with respect to the other one (*e.g.* Er^3+^ with respect to Tm^3+^).^6^ While sufficient to provide many colors, such probes are indistinguishable in homogeneous solutions because overlapping spectral bands are present in all labels (*e.g.* green 540 nm emission is present in Er^3+^ and Ho^3+^, and red 650 nm emission is present in Er^3+^, Ho^3+^, and Tm^3+^). In practical applications many nanometric labels co-localize spatially (*i.e.* within the point spread function volume) and spectrally (*i.e.* in optical band-pass filter based wide-field imaging), therefore spectral purity of luminescent labels is critical for multi-target biolabeling.^7^ While Er^3+^ and Tm^3+^ emission spectra are clearly different, the Er^3+^ and Ho^3+^ emission is spectrally similar. Although spectrally more unique,^8^ other lanthanides' emission intensity (*i.e.* Pr^3+^, Tb^3+^, Eu^3+^, and Sm^3+^) is typically too weak in upconversion mode to be applicable. Weak emission can be explained by high concentration quenching (*e.g.* Pr^3+^ and Sm^3+^) which limits the amount of activators and consequently limits emission intensity, or could be due to the energy upconversion mechanism other than energy transfer upconversion (ETU). The latter case is typical for Yb^3+^ sensitized Eu^3+^ and Tb^3+^ emission, which involves cooperative energy transfer (CET).^9–13^ These ions display unique and easily distinguishable spectral features, but CET is around 2–3 orders of magnitude less efficient in comparison to energy transfer upconversion found in the Yb^3+^–Tm^3+^/Er^3+^/Ho^3+^ pairs.^14^ For these reasons, terbium upconversion emission should be enhanced in order to provide labels with brightness comparable to that of Yb^3+^–Er^3+^ or Yb^3+^–Tm^3+^ in UCNPs and thus offer sufficient sensitivity for bioassays and bioimaging.

In our previous studies, we have proposed and demonstrated approaches to optimize the Yb^3+^–Tb^3+^ upconversion efficiency.^8,11,15^ Our results showed that up to 40-fold upconversion intensity enhancement may be obtained for the NaYF_4_:Yb^3+^/Tb^3+^ nanoparticles^8^ by NP surface passivation with an undoped shell compared to bare core NPs of the same composition. The enhancement of Tb^3+^ upconversion emission was also observed when thulium ions acting as a secondary sensitizer were admixed, since a more efficient energy transfer upconversion mechanism becomes involved (*i.e.* ETU instead of CET).^11,16,17^ In triply doped samples such as Tm^3+^–Yb^3+^–Tb^3+^, the emitting level of the Tb^3+^ level is populated by the cooperative process from ytterbium pairs and simultaneously through much more efficient energy transfer upconversion from excited thulium ions.^14,18^ Thulium ion co-doping allowed five-fold enhancement of the upconversion emission intensity from the ^5^D_4_ terbium level,^11^ but contaminated the emission spectrum of such labels with Tm^3+^ emission.

Recently, a new approach to the energy transfer between lanthanide ions and transition metal ions has also been investigated. Zhao's group demonstrated a new strategy for the rational manipulation of erbium green and red upconversion emission.^19^ They obtained pure red emission of NaYF_4_:Yb^3+^/Er^3+^ nanoparticles by manganese ion co-doping. The transition possibilities between green and red emissions of Er^3+^ were disturbed by the existence of Mn^2+^ ions leading to “concentrated” pure red emission.^19^ Because the energy transfer between Er^3+^ and Mn^2+^ is extremely efficient, as a result, single-band red upconversion emission was observed.^19–21^ Doping with Mn^2+^ ions has also been used to control the color of emission of NaYF_4_:Yb^3+^/Ho^3+^ nanocrystals to achieve enhanced red emission,^22,23^ and Zhang's group proposed an energy transfer mechanism from the Yb^3+^–Mn^2+^ dimer to Eu^3+^. The ^4^T_1_ energy level of Mn^2+^ matches well with the ^5^D_0_ energy level of Eu^3+^.^24–26^ Pure and intense orange upconversion luminescence of Eu^3+^ in NaY(Lu)F_4_ nanoparticles was obtained through the upconversion sensitization by the Yb^3+^–Mn^2+^ dimer. This is in stark contrast to Eu^3+^ upconversion achieved through Yb^3+^–Tm^3+^ upconversion, which concomitantly shows emission from both Tm^3+^ and Eu^3+^ ions.^27^ Such a new approach would conceptually be much more efficient than the Yb^3+^ → Tm^3+^ → Gd^3+^ → Eu^3+^ cascade and migration assisted ET in core–shell nanoparticles, although Liu *et al.* demonstrated it to be comparable or even higher in intensity than that in Yb^3+^/Er^3+^ co-doped nanoparticles.^6^

While energy transfer between Mn^2+^ and Tb^3+^ ions was investigated in various host matrixes,^28–31^ the UC emission intensity enhancement and Mn^2+^ sensitized ET processes between Tb^3+^ and Yb^3+^ co-doped NaYF_4_ nanocrystals have never been reported up to now. Tb^3+^ emission bands are located in the range from about 450 to 700 nm, whereas the Mn^2+^ broad emission band is located in the region from about 460 to 700 nm and strongly depends on the crystal environment of the host materials.^28,32^ Partial overlap of the emission bands confirms that energy transfer processes are likely to occur, and thus the major motivation for this research was to provide an additional possibility to enhance upconversion emission intensity of the Yb^3+^–Tb^3+^ co-doped β-NaYF_4_ nanocrystals through the sensitization by Yb^3+^ and Mn^2+^. To reach this goal, concentration dependent structural as well as spectroscopic studies have been performed which revealed the possibility of improving the UC efficiency. Additionally, the surface of the obtained nanoparticles was subsequently coated with proteins and conjugated with folic acid. Such prepared nanoparticles were used as an *in vitro* bioimaging agent. This is one of the very rare reports of cellular imaging experiments using Tb^3+^ pure upconversion emission,^33^ and the only one so far which demonstrates the applicability of core only β-NaYF_4_ colloidal nanoparticles doped with Tb^3+^ and Yb^3+^ ions in optical wide-field UC imaging.

## 2 Experimental

### 2.1 Materials

Yttrium oxide (99.99%), ytterbium oxide (99.99%), terbium oxide (99.99%), manganese(ii) carbonate, acetic acid (99%), pure oleic acid and 1-octadecene (90%), thiazolyl blue tetrazolium bromide (MTT), and Dulbecco's Modified Eagle's Medium (DMEM) were purchased from ALDRICH Chemistry. Ethanol (96% pure p.a.), methanol, *n*-hexane and chloroform were purchased from POCH S.A. (Poland). Fetal bovine serum (FBS), folic acid (FA) (≥97%) and dimethyl sulfoxide (DMSO) (≥99.5%) were purchased from Sigma Aldrich. *N*-Hydroxysuccinimide (NHS) (≥98%) and 1-ethyl-3-(3-dimethylaminopropyl)carbodiimide hydrochloride (EDC) (≥98%) were purchased from Alfa Aesar. Bovine serum albumin (BSA) (>98%) was purchased from BioShop. All of the chemical reagents were used as received without further purification.

### 2.2 Synthesis of nanoparticles

The hexagonal β-NaYF_4_:Mn^2+^,Yb^3+^,Tb^3+^ nanoparticles were prepared using a thermal decomposition reaction of lanthanide oleates.

#### Preparation of precursors (lanthanide acetate (CH_3_COO)_3_Ln and manganese acetate (CH_3_COO)_2_Mn)

Stoichiometric amounts of respective lanthanide oxides Y_2_O_3_, Tb_2_O_3_, and Yb_2_O_3_ and MnCO_3_ were mixed with 50% aqueous acetic acid. The mixture was stirred and heated to obtain a clear and transparent solution. The final precursor was obtained by evaporation of solvents in a pre-vacuum and further drying at 140 °C for 12 hours.

#### Preparation of the nanoparticles

The acetates [(CH_3_COO)_3_Y, (CH_3_COO)_3_Yb, (CH_3_COO)_3_Tb, and (CH_3_COO)_2_Mn] – 2.5 mmol – were added to a flask with 15 ml of oleic acid and 38 ml of octadecene. The solution was stirred and heated up to 140 °C under vacuum for 30 minutes to form an oleate complex and to remove total oxygen and remaining water. Next the temperature was lowered to 50 °C. 10 mmol ammonium fluoride (NH_4_F) and 6.25 mmol sodium hydroxide (NaOH) were dissolved in 20 ml of methanol and added to the reaction flask. The resulting mixture was stirred for 30 minutes at 70 °C. Next, the reaction temperature was increased and the methanol was evaporated. After removing the methanol, the solution was heated up to 300 °C under a nitrogen atmosphere and kept under such conditions for 1 hour. Next, the mixture was cooled to room temperature. The nanoparticles were precipitated using ethanol, centrifuged at 10 000 rpm for 10 minutes and washed with hexane and ethanol. Finally, the prepared nanoparticles were dispersed in chloroform. The colloidal solution was stable without aggregation.

### 2.3 Cell culture

The HeLa cell line was cultured in Dulbecco's Modified Eagle Medium (DMEM, Gibco) supplemented with 10% fetal bovine serum and an antibiotic–antimycotic solution (Sigma-Aldrich) containing penicillin (100 units per ml), streptomycin (100 μg ml^−1^) and amphotericin B (250 ng ml^−1^). Cells were incubated in a humidified incubator at 37 °C with 5% CO_2_.

### 2.4 Surface modification and functionalization of upconverting nanoparticles

200 μl of a chloroform suspension of oleic acid (OA) capped nanoparticles was added to 1 ml of 0.1 M HCl solution and vortexed for 20 minutes. The aqueous phase was placed into a new tube and centrifuged for 20 minutes (21 000 × *g*, RT). The pellet was subsequently washed with ethanol and 0.1 M HCl and suspended in 200 μl of DMSO (dimethyl sulfoxide). The suspension of nanoparticles was mixed with 1 ml of FBS (fetal bovine serum), incubated in an ultrasonic bath for 5 minutes and vortexed for 30 minutes. To separate the nanoparticles from FBS, the sample was repeatedly centrifuged (21 000 × *g*, 20 minutes, RT) and suspended in a solution of 0.9% NaCl and 1% BSA.

Folic acid was conjugated to the protein capped nanoparticles by the EDC/NHS chemistry. 2 mg of folic acid was activated by EDC and NHS in 140 μl of DMSO (the molar ratio of FA/EDC/NHS = 1 : 1 : 1). The solution was vortexed for 30 minutes at RT and then transferred dropwise to 1 ml of NP solution (4.25 mg ml^−1^) suspended in 0.1 M HBS (HEPES buffered saline), pH 8.0. The solution was vortexed for 3 hours at RT and then subsequently 100 μl of 1 M tris buffer, pH 9.0, and 200 μl of 1% BSA in saline were added to stop the reaction. Nanoparticles were purified by multiple centrifugation steps (21 000 × *g*, 20 minutes, RT) in saline and were finally suspended in 1 ml of 0.9% NaCl and 1% BSA and stored at 4 °C.

### 2.5 Fluorescence microscopy assay

For *in vitro* studies, 5 × 10^4^ HeLa cells were seeded on a glass-bottomed multiwell culture dish. After 8 h the cells were incubated with the FA-conjugated UCNPs for 20 h at 37 °C. Each well contained 400 μl of media with UCNPs at a concentration of 0.2 mg ml^−1^. To remove unbound UCNPs, the cells were washed 3 times with 0.9% NaCl and fixed with 3.7% formaldehyde solution in HBS for 10 min. Cells were washed 3 times with 0.9% NaCl and mounting media containing DAPI was added to label the nucleus.

### 2.6 Cytotoxicity of UCNPs

The cytotoxicity of NPs was evaluated using the MTT viability assay. The cells (4000 per well) were incubated in each well of a 96-well plate with β-NaYF_4_:5% Yb^3+^,15% Tb^3+^ (protein coated), β-NaYF_4_:5% Yb^3+^,15% Tb^3+^@FA (protein coated, with folic acid), β-NaYF_4_:10% Mn^2+^,5% Yb^3+^,15% Tb^3+^ (protein coated), and β-NaYF_4_:10% Mn^2+^,5% Yb^3+^,15% Tb^3+^@FA (protein coated, with folic acid) nanoparticles in the following concentrations: 50, 100, and 200 μg ml^−1^ in a medium (DMEM, 10% FBS) for 72 h. After that time, the cells were washed with PBS. Then the MTT solution (0.1 mg ml^−1^, DMEM w/o FBS) was added and the cells were incubated for 4 h. The MTT medium was removed from each well and the cells were washed with PBS. PBS was removed from each well, DMSO was added and the plates were shaken for 10 min at room temperature to dissolve the dye. The absorption was measured at *λ* = 570 nm in a microplate reader. The cell viability was calculated as follows: cell viability = *A*/*B* × 100%, where *A* is the absorbance of the cells incubated with DMEM containing NPs and *B* is the absorbance of the cells incubated with DMEM w/o NPs (as a control).

### 2.7 Characterization

Powder diffraction data were collected on an X'Pert PRO X-ray diffractometer equipped with a PIXcel ultrafast line detector, a focusing mirror and Soller slits for Cu Kα radiation. Transmission electron microscopy (TEM) and selected area electron diffraction (SAED) were performed on an FEI Tecnai G2 20 X-TWIN microscope operating at 200 kV. TEM-EDS investigations were carried out on a double-aberration corrected FEI Titan 60-300 cubed (S)TEM, operated at 300 kV. EDS (energy dispersive X-ray spectroscopy) spectra were acquired using Bruker ChemiSTEM super-X EDS detectors, with an acquisition time of 400 s. The photo-luminescence spectra were collected with a QE65000 high-sensitivity fiber optic spectrometer (OceanOptics) under 975 nm laser diode (*P*_max_ = 3W CW, LP975-3000 Spectra Laser, Poland) photo-excitation. Luminescence lifetimes were measured under 976 nm excitation with a CW 8.5 W laser diode modulated with a trigger from an FLS980 fluorescence spectrometer and with a PMT model R928P from Hamamatsu.

HeLa cell imaging was performed with an inverted fluorescence wide-field microscope, AxioObserverZ1 (Carl Zeiss) with an EC Plan-Neofluar 40×/1.3 oil DIC M27 objective with a condenser BF (NA = 0.4) to record the white light image, and 3 W CW 975 nm laser diode excitation (Spectra-Laser) in UC mode with a custom-mounted filter cube. The filter cube was composed of FF750-SDi02 dichroic and FF01-945 emission filters to cut out the 975 nm excitation and transmit visible radiation. To collect the images EMCCD Rolera EM-C2 (QImaging) as well as ZEN2011 software (Carl Zeiss) were used. To record the fluorescence images (*t* = 1/5 s, *f* = 2.8, ISO = 100) of the samples shown in Fig. 5 and 10b, a Canon 400D digital camera with a 60 mm *f* = 2.8 EF macro-lens (Canon) was used.

## 3 Results and discussion

The hexagonal β-NaYF_4_:Mn^2+^/Yb^3+^/Tb^3+^ nanoparticles were prepared using a thermal decomposition reaction of lanthanide oleates. In order to determine the impact of manganese ion concentration on the optical properties of the Tb^3+^–Yb^3+^ upconversion emission, nanoparticles with different concentrations of ions were synthesized. The concentration of nanoparticles in all samples was the same. This enables semi-quantitative comparison and characterization of all compounds in terms of morphology, structure and spectra. Different phenomena behind the expected upconversion enhancement may be preliminary proposed, such as the energy transfer process between Mn^2+^, Yb^3+^ and Tb^3+^ ions as well as the distortion of the crystalline field symmetry due to the introduction of Mn^2+^ ions replacing yttrium ions in the β-NaYF_4_ lattice. These two hypotheses will be further explored and evaluated.

### 3.1 Structural characterization of nanoparticles doped with Mn^2+^ ions

**Fig. 1:**
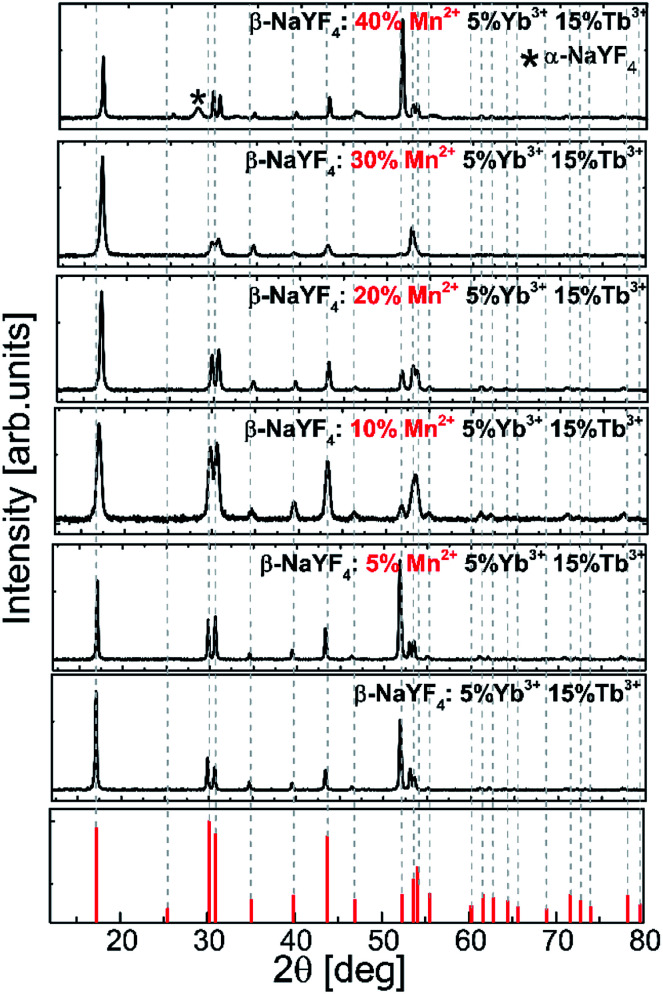
Experimental powder X-ray diffraction (XRD) patterns of the Tb^3+^, Yb^3+^, and Mn^2+^ codoped β-NaYF_4_ nanoparticles.

The influence of the Mn^2+^ ion concentration on the structure and the size of the nanoparticles was studied based on the powder X-ray diffraction patterns of β-NaYF_4_ (Fig. 1).

XRD reflection peaks of the nanoparticles are in agreement with the standard pattern of JCPDS card # 00-016-0334 and indicate a pure hexagonal phase. The hexagonal phase of NaYF_4_ still remains stable even when smaller Mn^2+^ (*r* = 0.81 Å) ions are introduced into the host lattices to replace larger Y^3+^ ions (*r* = 0.89 Å). Nevertheless, in the nanoparticles doped with Mn^2+^ at amounts higher than 30%, the XRD patterns showed a mixed phase of hexagonal and cubic NaYF_4_ phases.

**Fig. 2:**
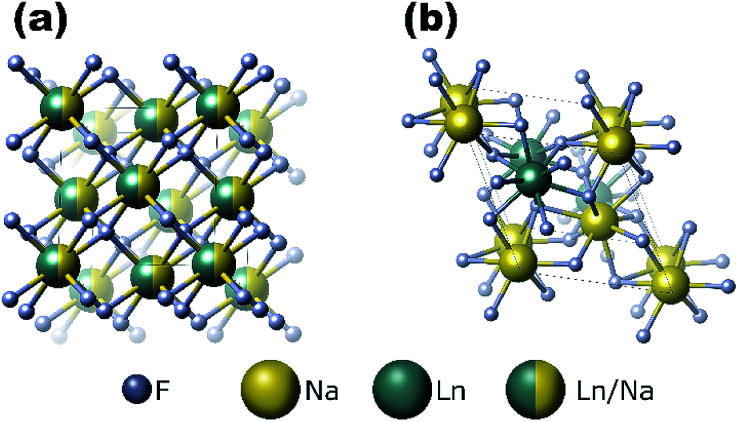
Schematic representations of (a) cubic- and (b) hexagonal-phase NaLnF_4_ structures.

The cubic structure (space group *Fm*3̄*m*) contains one type of cation site, and the lanthanide and sodium ions are equally and randomly distributed in the cationic sublattice (Fig. 2a). In the hexagonal structure, there are three types of cationic sites: (I) a 9-coordinated position occupied by Y^3+^, (II) a 9-fold coordinated position occupied randomly by 1/2 Na^+^ and 1/2 Y^3+^, and (III) a 6-fold coordinated position occupied by 1/2 Na^+^. The Y^3+^ sites are substituted randomly with other lanthanides ions.^34^ Previous reports have demonstrated that the size of the substitutional dopant ions plays a key role in stabilizing a specific crystalline phase in the NaYF_4_ structure. The substitution ions with a smaller ionic radius favor the cubic structure, whereas the larger substitution ions tend to produce the hexagonal phase of the final nanomaterials. Doping lanthanide ions with sizes larger than that of Y^3+^ in NaYF_4_ host lattices should lead to the dominant formation of pure hexagonal-phase NaYF_4_ nanocrystals.^35^

When the dopants have different valences than the substituted host ions, extra vacancies or interstitial ions are introduced into the host lattice during the nucleation and growth because of the charge compensation requirement. For hetero-valence ion doping, the variations of the crystal lattice are complex due to the requirement of extra vacancies or interstitial ions to compensate for the charge. When Mn^2+^ ions are doped into the crystal matrix, they may locate in either Ln^3+^ lattice sites or in interstitial positions. Doping additional ions into the crystal matrix induces a breakdown of the crystallographic site symmetry and may in consequence lead to phase transformation. The XRD pattern exhibits that the diffraction peaks of NaYF_4_:Tb^3+^,Yb^3+^ doped with different concentrations of Mn^2+^ ions shift toward higher 2*θ* values as the concentration of Mn^2+^ ions increases. It may result from the shrinkage of the unit-cell volume caused by substituting Y^3+^ ions with ions of a relatively smaller radius.^36^ This result is in good agreement with a previous report about Fe^3+^ and Cu^2+^ ion doped NaYF_4_ nanoparticles.^37^

**Fig. 3:**
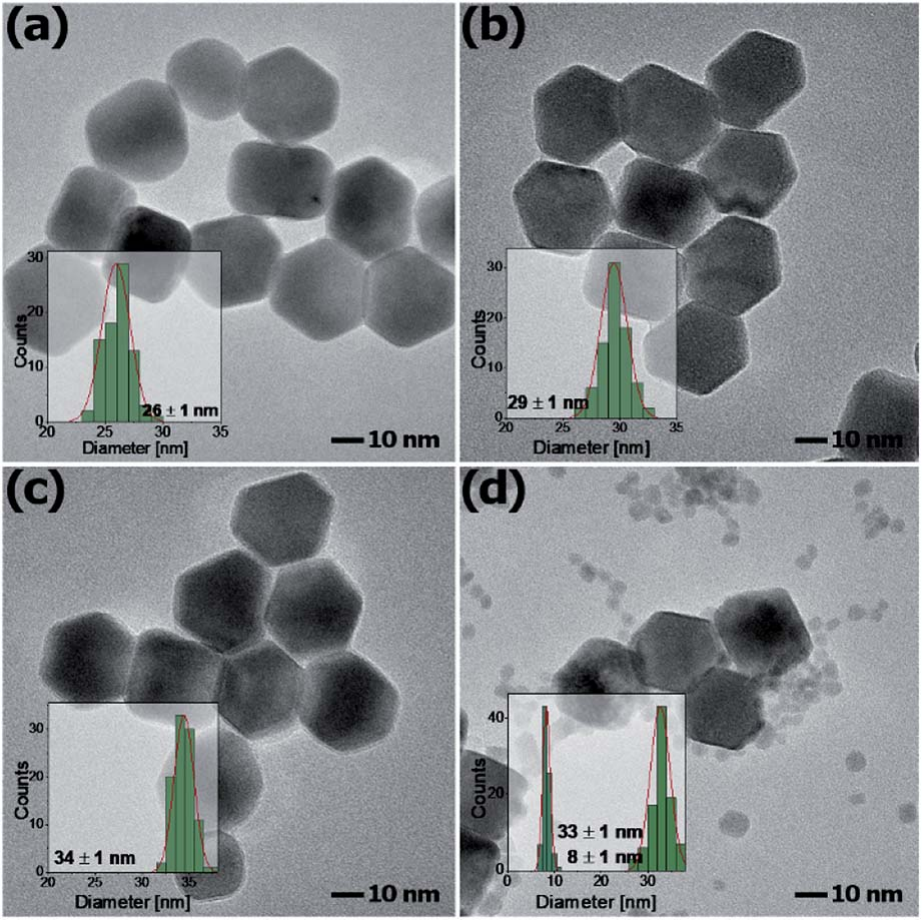
TEM images of β-NaYF_4_:Mn^2+^,Yb^3+^,Tb^3+^ nanocrystals doped with (a) 10%, (b) 20%, (c) 30% and (d) 40% Mn^2+^ ions.

Fig. 3 shows the representative TEM images of NaYF_4_:Mn^2+^/Yb^3+^/Tb^3+^ nanocrystals doped with different amounts of Mn^2+^ ions. Doping with Mn^2+^ ions caused a slight increase in nanoparticle size, but the NPs remained monodisperse. The average size for the Mn^2+^ un-doped sample can be estimated as *ca.* 23 nm, while for samples doped with 30% Mn^2+^ the nanoparticles grow to 33 nm. Further increasing the Mn^2+^ doping amount is detrimental for the quality of the samples, *i.e.* for the NaYF_4_:Mn^2+^/Yb^3+^/Tb^3+^ (40/5/15 mol%) samples, and the TEM images show two separate particle morphologies and sizes (Fig. 3d), which is consistent with the presence of two phases observed in the X-ray powder diffraction patterns (Fig. 1) exclusively for this sample. The small α-NaYF_4_ particles were irregular in shape and were *ca.* 8 nm in average size. These small NPs result from substitution of host ions by the smaller Mn^2+^ ions, which also tends to produce the cubic phase.^10^

**Fig. 4:**
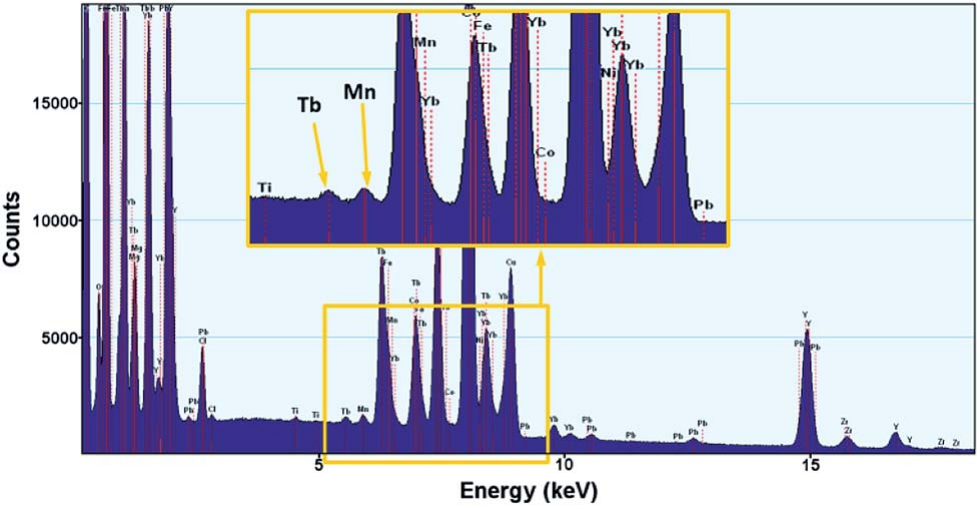
EDX spectrum of NaYF_4_ nanoparticles doped with Tb^3+^, Yb^3+^ and Mn^2+^ ions.

The presence of Mn ions (without determining the valence state) was clearly confirmed using EDX. Although many lines of Tb and Mn atoms overlap, we managed to confirm the presence of Mn ions – the K-a peak at 5.9 keV is unequivocal evidence of Mn presence within the nanocrystal structure (Fig. 4).

### 3.2 Optical properties of β-NaYF_4_:Mn^2+^,Yb^3+^,Tb^3+^ nanoparticles

**Fig. 5:**
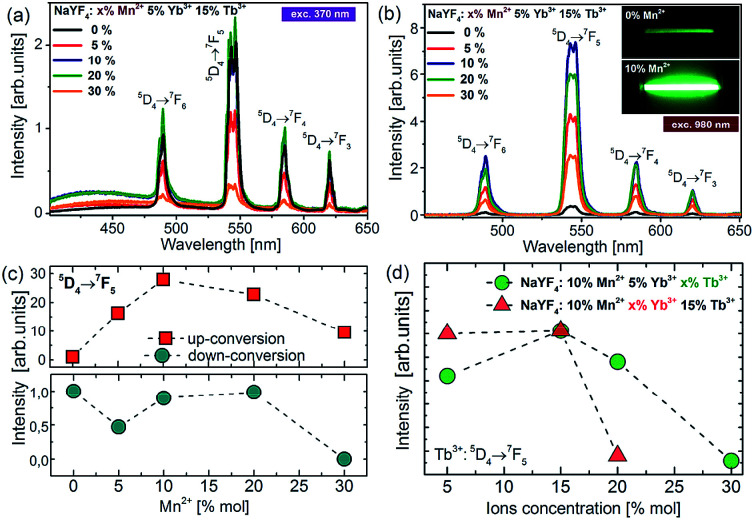
(a) The downconversion (DC) and (b) upconversion spectra of colloidal β-NaYF_4_:5% Yb^3+^,15% Tb^3+^ nanoparticles doped with different amounts of Mn^2+^ ions under 370 nm and 980 nm excitation, respectively. The inset shows the photographs of the upconversion luminescence from Mn^2+^ undoped (top) and co-doped (bottom) colloidal samples. (c) Terbium DC-emission and UC-emission intensity as a function of Mn^2+^ concentration. (d) Terbium UC emission intensity as a function of Yb^3+^ and Tb^3+^ ion concentration under 980 nm excitation.

To understand the actual processes responsible for the upconversion emission from Tb^3+^ in the presence of intentionally varied Mn^2+^ ion content, the photoluminescence spectra of colloidal nanoparticles were measured under UV (370 nm, Fig. 5a) and NIR (980 nm, Fig. 5b) excitation.

Upon the absorption of UV photons, Tb^3+^ ions are excited to their 4f^7^5d^1^ state, and non-radiatively relax down to the emitting ^5^D_4_ level. Owing to the energy gap law and low maximum available phonons, ∼350 cm^−1^ in the NaYF_4_ matrix, this process is not very efficient. Four distinct emission bands of Tb^3+^ were observed and could be assigned to the ^5^D_4_ → ^7^F_J_ (*J* = 3, 4, 5, and 6) transitions (Fig. 5a). For the nanoparticles doped with Mn^2+^ ions, a small broad emission band at 450 nm was observed, which most probably is due to the 4f^13^5d^1^–4f^14^ transition of the Yb^2+^ state. The broadband emission was similar to the one reported for NaYF_4_ nanoparticles doped with Tb^3+^, Yb^3+^ and Nd^3+^ ions in our previous work.^15^ Although Yb^2+^ broad band emission should typically occur at higher energy (*e.g.* in CaAl_2_O_4_, LiYF_4_, and CaF_2_) and at low temperatures, we observe a relationship between its intensity and the nanocrystal composition, which should not, in principle, take place, if the broad band emission arises only from the presence of organic ligands or other impurities on the surface of the nanocrystals. Nevertheless, the Yb^2+^ emission with the maxima at 440 nm has been observed in a few hosts, such as Sr_3_(PO_4_)_3_ at low temperatures, as well as the Yb^2+^ emission has been observed at room temperature from different matrices, showing a yellow-green-bluish colour with the maxima at ∼550 nm (in CaF_2_, b-SiAlON) or ∼430 nm (NaCl).

The quantitative comparison of photoluminescence properties of the as-prepared colloidal nanoparticles NaYF_4_:Tb^3+^,Yb^3+^ doped with Mn^2+^ has been presented in Fig. 5b. The nanocrystals exhibit relatively strong green upconversion emission under 980 nm NIR diode excitation. Three distinct emission peaks can be assigned to the ^5^D_4_ → ^7^F_6_ (*λ* = 480 nm), ^5^D_4_ → ^7^F_5_ (*λ* = 540 nm), ^5^D_4_ → ^7^F_4_ (*λ* = 585 nm) and ^5^D_4_ → ^7^F_3_ (*λ* = 620 nm) terbium transitions. As shown in Fig. 5, the presence of Mn^2+^ ions may significantly increase upconversion emission of Tb^3+^ ions. The most intense emission was achieved for nanoparticles doped with 10% Mn^2+^ ions, which was responsible for over 30 times brighter intensity of Tb^3+^ ions compared to the emission of nanocrystals without the addition of Mn^2+^ ions. A further increase of Mn^2+^ concentration caused a decrease in ^5^D_4_ → ^7^F_5_ transition intensity, but still the emission intensity was higher than for the samples without the addition of Mn^2+^ ions. Based on the result from upconversion spectra we can conclude that the concentration of Mn^2+^ ions should be kept similar to the amount of Yb^3+^ ions in nanocrystals.

To further optimize the UC intensity, besides Mn^2+^ optimization, additional nanocrystals with different Tb^3+^ and Yb^3+^ ion doping levels were also synthesized. The enhancement of Tb^3+^ emission intensity was obtained until 15 mol% Tb^3+^ ion doping, and then with the further increment of the doping amount, the intensity of green emission decreased, which most probably can be explained by the backward energy transfer from ^5^D_4_:Tb^3+^ ions to Mn^2+^ (Fig. 5d). A similar observation was made by Zhang's group in the case of nanoparticles doped with europium ions.^26^ They have studied the influence of Mn^2+^ ion doping on the luminescence properties of NaY(Lu)F_4_ nanoparticles doped with europium and ytterbium ions. They found that the optimal concentration of Eu^3+^ and Yb^3+^ ions lies in the range between 15% and 5%, respectively. Similarly in our materials, the optimal concentration of Yb^3+^ was estimated to be 5% because a further increase in Yb^3+^ concentration did not improve the UC emission intensity (Fig. 5d). We suppose that it can be linked with the high probability of occurrence of energy transfer to surface quenchers over long distances by energy migration through the Yb^3+^ network.^38^ Based on these results, the optimal concentration for effective energy upconversion transfer was estimated to be 15% Tb^3+^ and 5% Yb^3+^. In our previous work the optimum Tb^3+^ and Yb^3+^ concentration was estimated to be 40% and 20%, respectively,^8,11^ and the concentration ratio was equal to 2 : 1 previously and went up to 3 : 1 in this work. Obviously, this originates from Mn^2+^ ion co-doping; however, the exact mechanism of enhancement is not clear. Because the Tb^3+^ → Yb^3+^ energy back transfer is possible and significant energy migration to the surface quenchers at high Yb^3+^ doping is known,^39^ we may only speculate that the addition of Mn^2+^ ions not only supports more efficient CET, but also helps the Yb^3+^ network to enable more efficient energy migration and lose energy through the surface effects.

Despite the formation of Yb^3+^–Mn^2+^ dimers is well established in the literature,^40–43^ we have not managed to see broadband Mn^2+^ emission alone,^28,44^ which calls into question our preliminary hypothesis on the active participation of Mn^2+^ ions in the co-operative energy transfer in our nanoparticles (Fig. 7c). The hypothesis of cooperative sensitization of Mn^2+^ ions by a pair of Yb^3+^ ions, followed by ET to Mn^2+^ → Tb^3+^ (Fig. 7b) can also be proposed, but difficult to verify. In the view of the obtained results (*i.e.* mostly missing Mn^2+^ emission), the second, more probable explanation of the obtained enhancement of the upconversion emission can be proposed – *i.e.* the distortion of the crystalline field symmetry due to introduction of Mn^2+^ ions, replacing yttrium ions in the β-NaYF_4_ lattice (Fig. 7a). In the β-NaYF_4_ host lattice, the doped Mn^2+^ with a small radius can substitute cation ions or occupy interstitial sites, causing the shrinking or expansion of the host lattice and thus perturbing the symmetry of the local crystal field around Tb^3+^. The asymmetric surrounding environment emitting ions may lead to the faster energy transfer and in consequence increase the UC luminescence intensity.^45^ The maximum upconversion emission in the sample with 10% Mn^2+^ doping is attributed to the most asymmetric surrounding environment around Tb^3+^. With an increase of the doping concentration of Mn^2+^ from 0 to 30 mol%, the size of the nanoparticles increased monotonically, but their upconversion emission intensities increased at first and then decreased. One should also consider the fact that the size of nanoparticles increases along with the increasing Mn^2+^ content (Fig. 3) and by this indirect route, the Mn^2+^ ions could also enhance the CET emission. However, the increasing size does not correlate with the luminescence lifetimes of Yb^3+^ which become shorter with increasing Mn^2+^ levels. This observation should support the hypothesis of efficient Yb^3+^ → Mn^2+^ energy transfer, but in consequence, Mn^2+^ green and broadband emission should simultaneously be observed, which is not the case.

**Fig. 6:**
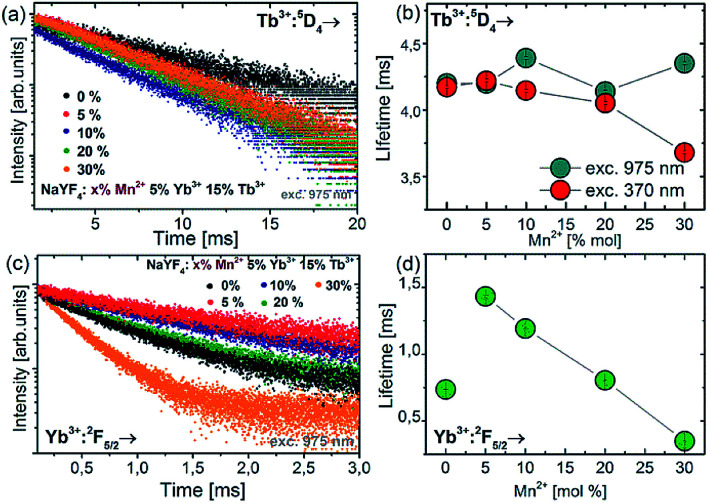
Luminescence decay curves of the ^5^D_4_ energy level of Tb^3+^ ions (a) and Yb^3+^:^7^F_5/2_ level (c) for NaYF_4_ colloidal nanoparticles doped with different amounts of Mn^2+^ ions. Concentration dependence of luminescence lifetimes for Tb^3+^:^5^D_4_ (b) and Yb^3+^:^2^F_5/2_ (d) energy levels.

In order to study the upconversion emission mechanism and to investigate the impact of the Mn^2+^ ion doping, the decay profiles of Tb^3+^ and Yb^3+^ emission were measured. The examples of ^5^D_4_ luminescence decay curves and luminescence decay values for different amounts of Mn^2+^ ions are presented in Fig. 6.

Doping of NaYF_4_:Tb^3+^,Yb^3+^ nanoparticles with Mn^2+^ ions did not significantly change the luminescence lifetime of the ^5^D_4_ energy level (measured at 975 nm excitation wavelength). In contrast to 975 nm excitation wavelength, increasing the Mn^2+^ concentration resulted in a luminescence lifetime decrease at 370 nm excitation, from *ca.* 4.3 ms down to the shortest value of 3.6 ms for NaYF_4_:5% Tb^3+^,15% Yb^3+^,30% Mn^2+^. The measurements of decay time were also carried out for the Yb^3+^ ions under 975 nm excitation. The luminescence lifetimes of the Yb^3+^:^2^F_5/2_ level decreased significantly as well when Mn^2+^ concentration was increased. Luminescence decays for Yb^3+^ were found to decrease monotonically with increasing Mn^2+^ concentration, which is evidence for efficient energy transfer from Yb^3+^ to Mn^2+^ ions.

**Fig. 7:**
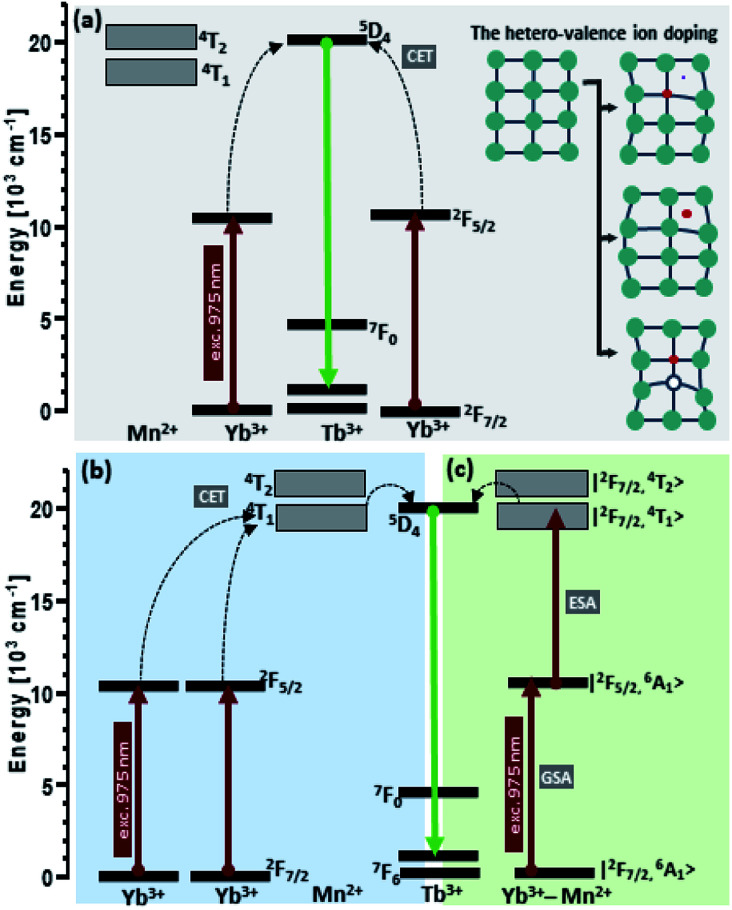
Energy transfer mechanisms of NaYF_4_:Mn^2+^,Tb^3+^,Yb^3+^ nanocrystals under 975 nm excitation. The obtained enhancement of the upconversion emission intensities can potentially result from (a) the tailored local crystal field around the Tb^3+^ ions induced by the unit cell expansion; (b) cooperative sensitization of Mn^2+^ ions by a pair of Yb^3+^ ions, followed by ET to Mn^2+^ → Tb^3+^ and Mn^2+^–Yb^3+^ “dimer” formation.^40–42^

Because we were not able to confirm unambiguously what is the reason for the observed increase in the intensity of terbium ion emission, three hypothetical upconversion mechanisms were discussed as shown in Fig. 7. Conventionally, for Tb^3+^ and Yb^3+^ ion doped nanocrystals under NIR photoexcitation at ∼980 nm, the ^5^D_4_ level population originates from cooperative energy transfer (cooperative sensitization) from two excited ytterbium ions (Yb^3+^–Yb^3+^ pair).^11,46^ Addition of Mn^2+^ ions may change the rate of energy transfer resulting from the tailored local crystal field around the active ions induced by perturbations of the symmetry of the local crystal field (Fig. 7a).^47^ At the same time, either the cooperative sensitization process of Mn^2+^ ions by two Yb^3+^ ions^28^ (Fig. 7b) or Mn^2+^–Yb^3+^ “dimer” formation^40–42^ (Fig. 7c) hypotheses have been postulated. This is achieved by combining the energy of two excited Yb^3+^ ions by a cooperative process to transfer it to Mn^2+^ ions. Next, the energy is transferred from the Mn^2+^ ions to the ^5^D_4_ state of Tb^3+^ ions.^28,48^ From the Tb^3+^:^5^D_4_ level, the energy can be transferred to the ^7^F_J_ levels and generate luminescence. Because we did not see concurrent Mn^2+^ emission (which should occur in the green spectral region), we could not confirm that Mn^2+^ ions contribute to upconversion energy transfer processes actively and directly, *i.e.* through the [Yb^3+^–Yb^3+^] → Mn^2+^ → Tb^3+^ or [Yb^3+^–Mn^2+^] → Tb^3+^ processes, respectively. Quenching of Tb^3+^ emission and Yb^3+^ lifetimes by increasing Mn^2+^ content suggests that Tb^3+^ → Mn^2+^ and Yb^3+^ → Mn^2+^ should be possible, but also here, Mn^2+^ emission was not found.

All these facts suggest that the passive role of Mn^2+^ co-doping dominates over detrimental quenching by Mn^2+^ ions, which led to the observed over 30-fold UC enhancement. Such an enhancement is, however, sufficient to make Tb^3+^ upconversion comparable to conventional Er^3+^ upconversion and can be used in biomedical imaging.

### 3.3 Bioimaging

Due to the hydrophobic nature of the capping ligand (oleic acid), nanoparticles’ surface is hydrophobic. Further functionalization is necessary to make the nanoparticles water dispersible and to use the synthesized nanocrystals as imaging labels.

**Fig. 8:**
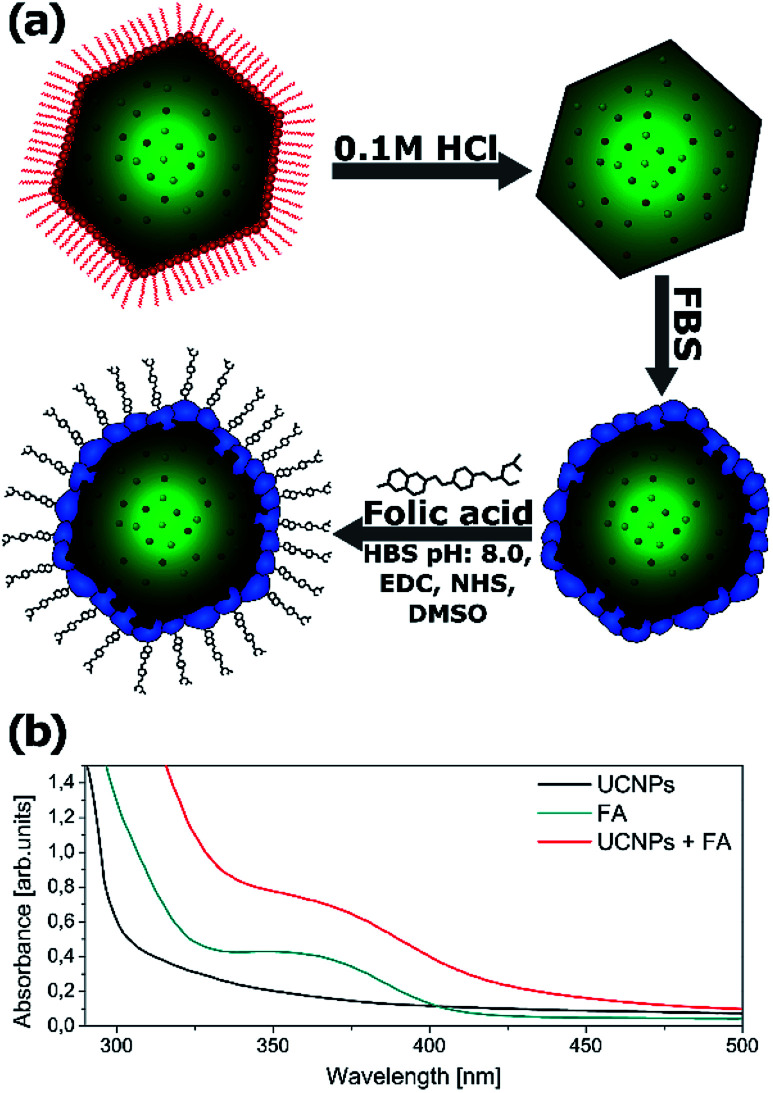
(a) Schematic illustration of the simple method used for the biofunctionalization of upconverting nanocrystals. (b) The UV-Vis absorption spectra of FBS-coated UCNPs before and after folic acid (FA) conjugation. UCNPs (0.5 mg ml^−1^), UCNP–FA (0.5 mg ml^−1^) and FA (50 μg ml^−1^) were dissolved in 0.1 M HEPES, pH 8.

Water dispersible nanoparticles were achieved by removing the oleic acid molecules from the surface of the nanocrystals at low pH using 0.1 M hydrochloric acid.^49^ The hydrophilic nanoparticles were mixed with FBS (fetal bovine serum) in order to initialize the formation of a protein corona on the nanoparticles’ surface.^50^ To make the synthesized nanoparticles suitable for cell imaging, the NPs were further biofunctionalized with folic acid (FA) (Fig. 8a). Both Yb^3+^/Tb^3+^ and Yb^3+^/Tb^3+^/Mn^2+^ UCNPs were treated with exactly the same protocol.

To confirm that the biofunctionalization was successfully performed and the folic acid is present on the nanoparticles’ surface, the absorption spectra of the NPs were measured after washing the free FA (Fig. 8b). The absorption spectra of the UCNPs changed after FA conjugation and exhibited an additional peak corresponding to the FA absorbance spectrum indicating successful conjugation of FA with NaYF_4_ nanoparticles. A similar absorbance change was reported by Hu and coworkers after folic acid conjugation to gold nanoclusters.^51^

To test the potential biological application of nanocrystals doped with Tb^3+^ ions, which offer spectrally distinguishable “fingerprints” compared to other dopants (*e.g.* Er^3+^ and Ho^3+^), *in vitro* bioimaging of the HeLa cell line was performed. HeLa is the first immortal cell line, established in 1952 by George Otto Gey. It derives from a cervical cancer patient, Henrietta Lacks, who died in 1951. Over the years, it became a model cell line in molecular cell biology, used in many publications and contributed greatly to the understanding of the biology of the human cell, cancer research and development of different vaccines.^52^ This particular cell line was selected due to its relatively fast growth rate with the cell count doubling in about 22 hours,^53^ as well as increased expression of folate receptors which is also a common feature of many other cancer cell types.^54^ The folate receptor effectively mediates the delivery of folate conjugated compounds and nanoparticles *via* receptor mediated endocytosis.^55^

**Fig. 9:**
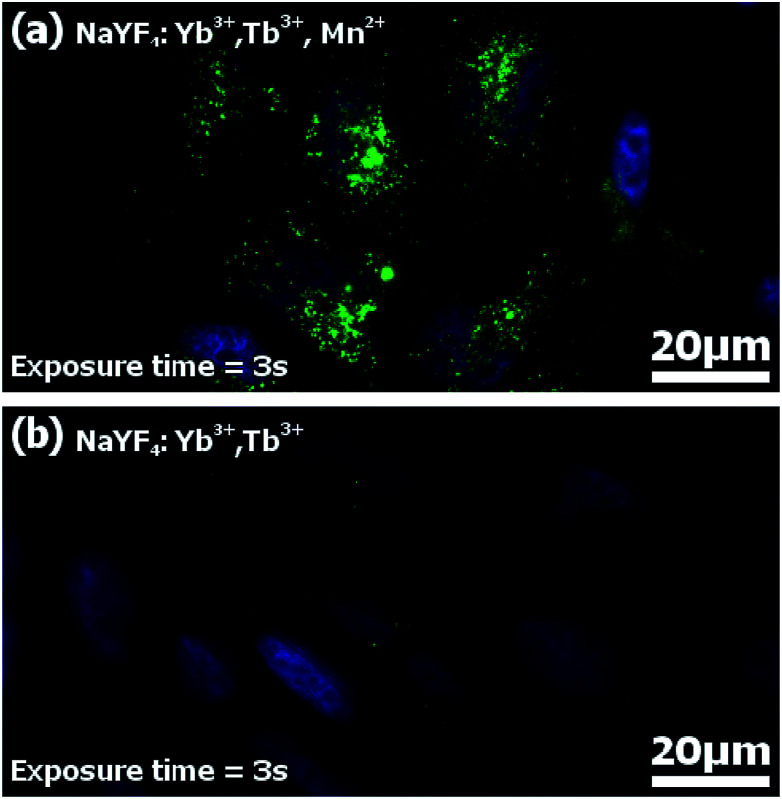
The upconversion and DAPI microscopy images (40× magnification, UV and 975 nm excitation) of HeLa cells after incubation with folic acid-conjugated (a) NaYF_4_:Yb^3+^,Tb^3+^, Mn^2+^ – exposure time 3 seconds in UC mode, and (b) NaYF_4_:Yb^3+^,Tb^3+^ – exposure time 3 seconds in UC mode. Cells were incubated for 20 hours with UCNP–FA (0.2 mg ml^−1^). Green colour represents upconversion fluorescence, and blue colour – fluorescence from nucleic acid stained with DAPI (4′,6-diamidino-2-phenylindole dihydrochloride).

Imaging of HeLa cells was done by incubation of the cells with FA-conjugated NaYF_4_:Yb^3+^,Tb^3+^,Mn^2+^ and NaYF_4_:Yb^3+^,Tb^3+^ nanocrystals. Functionalized nanoparticles were effectively internalized by HeLa cells upon 20 hours of incubation. The visible upconversion fluorescence of the NaYF_4_ nanocrystals doped with Mn^2+^, Tb^3+^, and Yb^3+^ ions loaded in HeLa cells was easily observed following excitation with 980 nm NIR radiation (Fig. 9a). The UC images were taken with 3 second exposure time. For nanocrystals without Mn^2+^ ions, the control luminescence imaging measurements under the same experimental conditions (the same camera setting and exposure time of 3 seconds) (Fig. 9b) showed significantly weaker emission than for the imaging of Mn^2+^ doped nanoparticles (Fig. 9a and b). Increasing the UC imaging acquisition time up to 30 seconds did not radically help to achieve any better signal from the sample, and only barely visible green upconversion emission was observed (not shown). Significant differences in the fluorescence intensity between both types of nanoparticles combined with the same surface bio-chemistry clearly demonstrated the advantages of nanoparticles doped with additional Mn^2+^ ions.

While the imaging in upconversion mode is nothing extraordinary with Yb^3+^/Er^3+^ or Yb^3+^/Tm^3+^ UCNPs, the UC imaging with Yb^3+^/Tb^3+^ codoped NPs has been severely hindered so far because CET (*e.g.* Yb^3+^/Tb^3+^) is typically 2 orders of magnitude weaker than ETU (*e.g.* Yb^3+^/Er^3+^ and Yb^3+^/Tm^3+^).

**Fig. 10:**
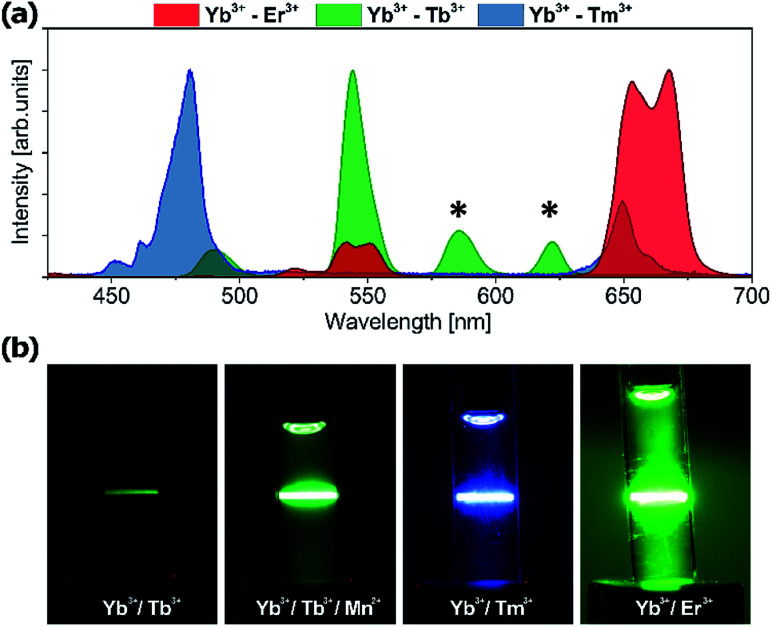
(a) Upconversion spectra of nanocrystals doped with Yb^3+^/Er^3+^, Yb^3+^/Tm^3+^ and Yb^3+^/Tb^3+^ ions. (b) Photographs of the upconversion luminescence from colloidal NaYF_4_ nanoparticles doped with different lanthanide ions measured at the same concentrations and with the same recording settings.

Tb^3+^ emission is easily distinguishable from Tm^3+^ and Er^3+^ emission because of the spectrally distinct 580 nm Tb^3+^ emission band. Upconversion spectra of Yb^3+^/Er^3+^, Yb^3+^/Tm^3+^ and Yb^3+^/Tb^3+^ codoped NaYF_4_ nanoparticles and the photographs of the upconversion luminescence from colloidal NaYF_4_ doped with different lanthanide ions are presented in Fig. 10. The Tb^3+^ emission bands marked by an asterisk are spectrally distinguishable from the emission of other lanthanide ions, despite some spectral overlap exists between Tb^3+^ and Er^3+^ or Tb^3+^ and Tm^3+^ at other wavelengths (*e.g.* 540 or 650 nm). However, because the emission branching ratio of Tb^3+^ emission is fixed, *i.e.* the relative proportions of ^5^D_4_ → ^7^F_J_ (*J* = 6.0) remain constant, knowledge of this branching ratio and the intensity at 580 nm enable us to appropriately correct 540 nm and 650 nm emission intensity, aiming at quantitative analysis of multiple labels, which overlap in space. The application of other nanomaterials doped with Tb^3+^ ions in bioimaging has been investigated with promising results but usually under UV excitation.^56–59^ However, UV photoexcitation demonstrates well known drawbacks such as generation of autofluorescence from endogenous intracellular fluorophores (*e.g.* aromatic groups of protein amino acids, nucleic acids, and low molecular coenzymes like porphyrins, NADH, FAD, *etc.*),^60^ which results in an undesirably high background and a low signal to background ratio. Moreover, at near-UV excitation wavelengths the penetration depth of light is limited to less than 200 μm for the human skin which is due to the presence of many UV-absorbing elements and increased light scattering in the heterogeneous tissue.^61^ Finally, UV poses some risk of damaging biomolecules, being the result of highly reactive free radical generation by UV radiation, or disruption of DNA integrity *via* breaking the hydrogen bonds between DNA strands.^62^

In this respect the use of NIR photoexcitation and upconversion emission is advantageous because it enables signal detection without an autofluorescence background and with limited light scattering, as biomolecules are usually much less efficient non-linear emitters (*e.g.* in 2- or 3-photon excitation mode). Moreover, the infrared wavelength provides a much deeper penetration depth of the optical radiation into samples up to 2 cm.^63^

### 3.4 Cytotoxicity

**Fig. 11:**
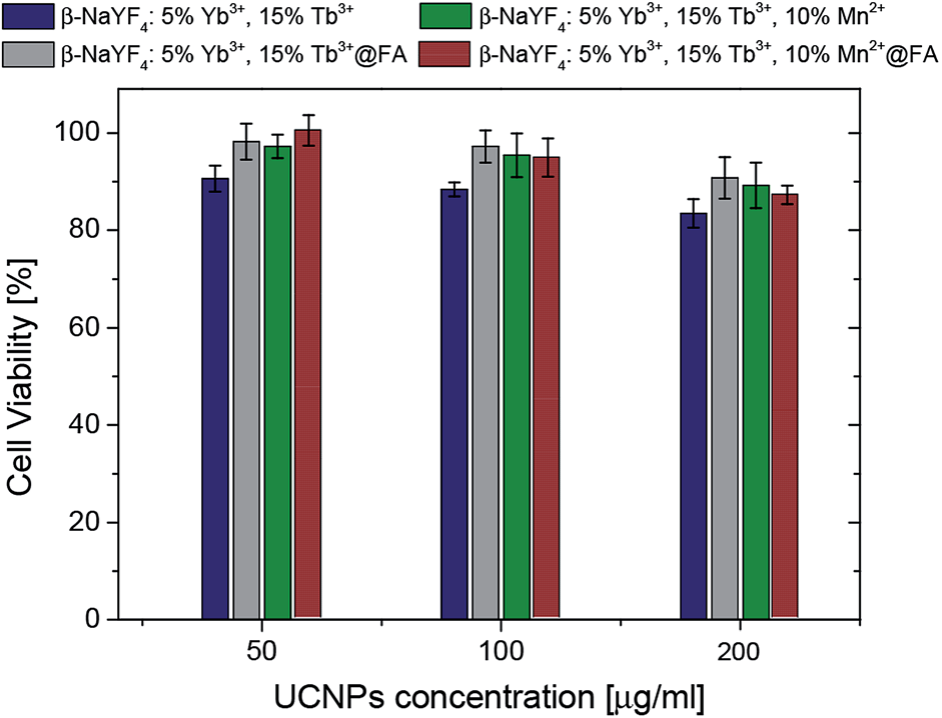
The viability of HeLa cells after 72 h of incubation with NaYF_4_ nanoparticles.

In order to investigate the cytotoxicity of the synthesized nanoparticles, an MTT assay using HeLa cells was performed. The impact of the UCNPs on the cell proliferation after 72 hours of incubation was studied (Fig. 11).

In all bio-applications, the important potential risk of the nanoparticles is their cytotoxicity. Chemical compositions of nanoparticles as well as their surface modifications play important roles in this matter. For these reasons, the MTT assay was used to investigate the cytotoxicity of the following UCNPs: β-NaYF_4_:5% Yb^3+^/15% Tb^3+^ (protein coated), β-NaYF_4_:5% Yb^3+^/15% Tb^3+^@FA (protein coated, with folic acid), β-NaYF_4_:10% Mn^2+^/5% Yb^3+^/15% Tb^3+^ (protein coated), β-NaYF_4_:10% Mn^2+^/5% Yb^3+^/15% Tb^3+^@FA (protein coated, with folic acid). No significant difference in the cell viability was observed in the presence of 50–200 μg ml^−1^ UCNPs (Fig. 11). The viability of the cells was estimated to be greater than 80% for β-NaYF_4_:5% Yb^3+^/15% Tb^3+^ and greater than 90% for β-NaYF_4_:5% Yb^3+^/15% Tb^3+^@FA, β-NaYF_4_:10% Mn^2+^/5% Yb^3+^/15% Tb^3+^ and β-NaYF_4_:10% Mn^2+^/5% Yb^3+^/15% Tb^3+^@FA after 72 hours of incubation. The data show that all the examined NPs, with a modified surface, have low cytotoxicity even at a relatively high concentration (200 μg ml^−1^), which is in agreement with a large number of reports on the toxicity of lanthanide doped NaYF_4_ UCNPs.^64^

## 4 Conclusion

β-NaYF_4_:Yb^3+^,Tb^3+^ nanocrystals doped with different concentrations of Mn^2+^ ions were synthesized in response to the demand for novel UC enhancement methods of weak Yb^3+^–Tb^3+^ upconversion. The significant enhancement of the UC emission as a result of the introduction of Mn^2+^ ions was observed without any changes in the crystallographic phase. The upconversion emission intensity obtained from the nanoparticles co-doped with 10% Mn^2+^ ions increased up to 30-fold in comparison to the corresponding nanoparticles without Mn^2+^ ions. Three different energy transfer mechanisms of the UC process were originally proposed, but following the analysis of the experimental results, we suppose that the obtained enhancement of the upconversion emission intensities can result from the tailored local crystal field around the Tb^3+^ ions being induced by the unit cell expansion as well as potentially an influence of the manganese ions acting actively in the transfer of energy.

The studies of the optimized Yb^3+^–Tb^3+^ UC based on Mn^2+^ enhanced upconversion are important for numerous reasons. First, the Yb^3+^ → Mn^2+^ → Tb^3+^ is a direct type of energy transfer which does not require more complicated indirect Yb^3+^ → Tm^3+^ → Tb^3+^ or Yb^3+^ → Tm^3+^ → Gd^3+^ → Tb^3+^ energy migration mediated upconversion, where core–(multiple)shell NPs have to be synthesized. Moreover, no spectral features originating from Tm^3+^ emission are present in the direct Tb^3+^ upconversion. In consequence, direct ETU based materials are also important when the size of the NPs is critically important, because homogeneously doped NPs can be smaller and much easier to obtain than core–shell NPs. As a proof of the performed enhancement, wide field UC imaging and visualization of folate-mediated endocytosis in FA receptor rich HeLa cells were successfully carried out, unlike with un-optimized Yb^3+^/Tb^3+^ UCNPs. Finally, a great advantage of labels doped with Tb^3+^ ions is the fact that their emission at 580 nm is spectrally easily distinguishable from the UC emission of conventionally used Tm^3+^ and Er^3+^ ions, which increases the selection of unique labels within the highly photostable, upconverting class of labels.

## Conflicts of interest

There are no conflicts to declare.

## Supplementary Material
